# Probiotic strain *Lactobacillus plantarum* 299v increases iron absorption from an iron-supplemented fruit drink: a double-isotope cross-over single-blind study in women of reproductive age

**DOI:** 10.1017/S000711451500241X

**Published:** 2015-10-28

**Authors:** Michael Hoppe, Gunilla Önning, Anna Berggren, Lena Hulthén

**Affiliations:** 1 Department of Gastroenterology and Hepatology, Section for Clinical Nutrition, Sahlgrenska University Hospital, Gothenburg, Sweden; 2 Probi AB, Ideon Gamma 1, Lund, Sweden; 3 Biomedical Nutrition, Pure and Applied Biochemistry, Centre for Applied Life Sciences, Lund University, Lund, Sweden; 4 Department of Internal Medicine and Clinical Nutrition, Sahlgrenska Academy at the University of Gothenburg, Gothenburg, Sweden

**Keywords:** Probiotics, *Lactobacillus plantarum* 299v, Fruit drinks, Iron status, Radio iron, Radioisotopes, Females

## Abstract

Iron deficiency is common, especially among young women. Adding probiotics to foods could be one way to increase iron absorption. The aim of this study was to test the hypothesis that non-haem iron absorption from a fruit drink is improved by adding *Lactobacillus plantarum* 299v (Lp299v). Iron absorption was studied in healthy women of reproductive age using a single-blind cross-over design in two trials applying the double-isotope (^55^Fe and ^59^Fe) technique. In Trial 1, iron absorption from a fruit drink containing 10^9^ colony-forming units (CFU) Lp299v was compared with that from a control drink without Lp299v. Trial 2 had the same design but 10^10^ CFU were used. The test and control drinks contained approximately 5 mg of iron as ferrous lactate and were labelled with ^59^Fe (B) and ^55^Fe (A), respectively, and consumed on 4 consecutive days in the order AABB. Retention of the isotopes was measured with whole-body counting and in blood. Mean iron absorption from the drink containing 10^9^ CFU Lp299v (28·6(sd 12·5) %) was significantly higher than from the control drink (18·5(sd 5·8) %), *n* 10, *P*<0·028). The fruit drink with 10^10^ CFU Lp299v gave a mean iron absorption of 29·1(sd 17·0) %, whereas the control drink gave an absorption of (20·1(sd 6·4) %) (*n* 11, *P*<0·080). The difference in iron absorption between the 10^9^ CFU Lp299v and the 10^10^ CFU Lp299v drinks was not significant (*P*=0·941). In conclusion, intake of probiotics can increase iron absorption by approximately 50 % from a fruit drink having an already relatively high iron bioavailability.

Iron deficiency and low iron status are common all around the globe^(^ ^1^ ^)^, and women of reproductive age are a vulnerable population because of their high iron requirements^(^ ^2^ ^,^ ^3^ ^)^. Besides inadequate iron intake, low iron bioavailability is the predominant reason for iron deficiencies in populations subsisting on plant-based diets, independent of sex^(^ ^4^ ^)^. Strategies to increase the intake of foods rich in iron, as well as dietary factors with enhancing effect on iron absorption, are therefore important.

A number of dietary factors affect the absorption of non-haem iron. Intake of ascorbic acid and meat stimulates absorption, whereas calcium, polyphenols (e.g. in tea, coffee, vegetables) and phytates (e.g. whole grain cereals) inhibit absorption^(^ ^5^ ^)^. Lactic acid-fermented foods may also improve the non-haem iron absorption. A number of single-meal studies with fermented vegetables and cereals have shown a significant increase in iron absorption in humans^(^ ^6^ ^–^ ^8^ ^)^. Lactic acid-fermented foods can increase iron absorption in humans, possibly by lowering pH, activating phytases, producing organic acids or by the viable lactic acid bacteria.

The strain *Lactobacillus plantarum* 299v (Lp299v) has been shown to survive the passage through the gastrointestinal tract irrespective of gastric acidity^(^ ^9^ ^)^. The strain colonises the intestine and can be found in mucosal samples taken from the jejunum and rectum 11 d after administration^(^ ^10^ ^)^. In clinical studies, Lp299v has been shown to have a positive effect on health. Effects include reducing gas problems and pain^(^ ^11^ ^–^ ^13^ ^)^ in people who suffer from irritable bowel syndrome. Intake of Lp299v can also counteract certain unwanted bacteria in the intestine^(^ ^14^ ^)^, and it can also have an anti-inflammatory effect by reducing the content of fibrinogen, ROS and IL-6 in the serum of subjects in a proinflammatory state^(^ ^15^ ^,^ ^16^ ^)^. An earlier study showed that intake of Lp299v can increase iron absorption by over 100 % from an oat base with low iron bioavailability (contains high levels of phytic acid (PA))^(^ ^17^ ^)^. The iron absorption was very low (1·1 % of the non-haem iron), and it is not known whether Lp299v can affect iron absorption in a meal with a higher availability of iron, thereby justifying the present study.

The primary aim of the study was to test the hypothesis that non-haem iron absorption from a fruit drink is improved by the addition of Lp299v. The secondary aim was to investigate whether this hypothesised effect is dependent on the Lp299v concentration by giving the subjects a dose of 10^9^ or 10^10^ CFU Lp299v.

## Methods

### Design

The study comprised two cross-over double radio-iron isotope single-blinded trials in young healthy Swedish women. The reason for confining the study to women was that women are one of the main vulnerable populations when it comes to iron deficiency. This double radio-iron method enabled the effect of adding or not adding Lp299v on iron absorption to be measured separately in the same individual. The two separate trials were performed from August to December 2007.

### General protocol

**Fig. 1.**
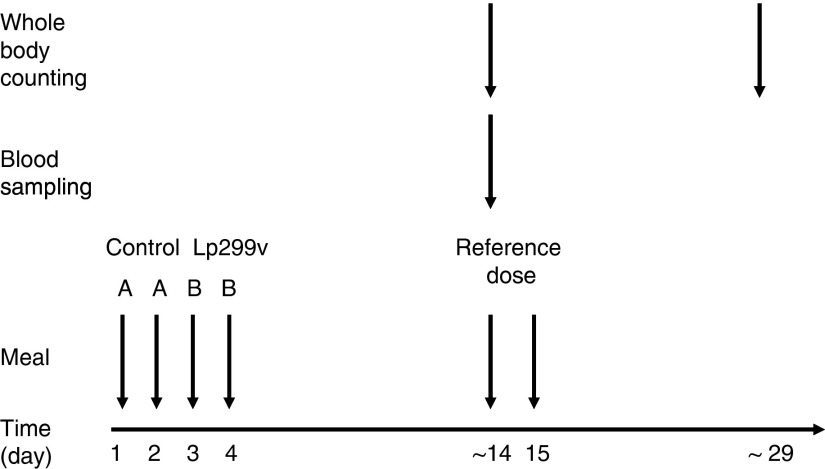
Study design. Fruit drink A (with ^55^Fe, but without Lp299v) and B (with ^59^Fe and Lp299v) were administered on 4 consecutive days. Approximately 2 weeks later, a whole-body counting was performed in order to assess the total body iron retention from the *γ*-emitting ^59^Fe (B). Directly after, a blood sample was drawn, in which the relative absorption of the two isotopes was assessed, after which a reference dose containing ^59^Fe-labelled ferrous iron was administered. A reference dose was also administered the following day. Two weeks later the absorption from the reference dose was measured in the whole-body counter.

In each trial, eleven subjects were served either a 200-ml fruit drink containing Lp299v (B) or a 200-ml fruit drink without lactobacilli (A). In Trial 1, drink B contained 10^9^ colony-forming units (CFU) Lp299v, and in Trial 2 it contained 10^10^ CFU Lp299v. The fruit drink was served as breakfast during 4 consecutive days in the order AABB. During the first 2 d, the drink was served without lactobacilli, after which the fruit drink containing lactobacilli was served on days 3 and 4. This order was chosen to avoid the possible effect of any lactobacilli remaining in the body if Lp299v was administered before the placebo. No food or drink was allowed within 3 h of the intake of the fruit drink. To determine the amount of iron absorbed, the iron in the fruit drinks was homogeneously labelled with extrinsic ^59^Fe (B) and ^55^Fe (A). Each portion of fruit drink was labelled, through pipetting, with the radioisotope (as FeCl_3_) immediately before serving. Blood sample was taken approximately 2 weeks after fruit drink administration. See Fig. 1 for study design.

### Measurement of iron absorption

The double-isotope technique has been used in human studies for 50 years and is well validated^(^ ^18^ ^,^ ^19^ ^)^. The basis for this methodology lies in the ‘pool concept’^(^ ^20^ ^,^ ^21^ ^)^, which is based on the diffusion-driven isotopic exchange occurring between extrinsically added iron and native iron in a meal – that is the common available pool of iron. In short, iron absorption is assessed by calculating the difference between the administered radioactivity and the radioactivity measured in the body. One of the advantages of using two different isotopes is that each person becomes their own control.

Each drink was administered on an empty stomach (i.e. no food after 22.00 hours and no drink after 24.00 hours the evening before) over consecutive days, after which the mean daily iron absorption was calculated. This was to reduce the effect of day-to-day variation in iron absorption.

Approximately 2 weeks (10–16 d) after drink administration, a whole-body counting was performed using a very sensitive whole-body counter^(^ ^22^ ^)^.

The iron absorption from the ^59^Fe-labelled fruit drink (B) was calculated as the percentage of detected whole-body radioactivity, corrected for physical decay and background radioactivity. The radiation from ^59^Fe corresponds to the amount of absorbed ^59^Fe, which in turn, according to the ‘pool concept’, corresponds to the amount of absorbed total iron from the drink containing Lp299v. However, absorption from ^55^Fe (i.e. the drink without Lp299v) cannot be detected by whole-body counting. Consequently, after the whole-body counting, a blood sample was drawn in which the relative absorption of each of the two isotopes was determined using a liquid scintillator. This relative absorption was then used, together with the blood volume calculated from each individual’s height, weight and Hb concentration^(^ ^23^ ^)^, to also calculate the total body ^55^Fe absorption (i.e. fruit drink A).

After the whole-body counting and the blood test, a reference dose (10 ml of 0·01 mol HCl containing 3 mg of ^59^Fe-labelled iron (II)+30 mg of ascorbic acid) was administered orally as breakfast together with 100 ml of water. Another reference dose was administered as breakfast the following morning. No food or drink was allowed within 3 h after the intake of each of the reference doses. The absorption from the reference dose was measured after another 2 weeks in the whole-body counter. By relating the absorption from the fruit drinks to this reference dose absorption, the variation depending on differences in iron absorption capacity was corrected.

The recorded radioactivity for each subject in the different trials amounted to a total of 2 μCi from ^55^Fe and 2·0 μCi from ^59^Fe (2×0·5 μCi from reference dose+2×0·5 μCi from fruit drink). A wet chemical analysis of ^55^Fe and ^59^Fe was carried out according to a modification of the analysis method described by Eakins & Brown^(^ ^24^ ^)^. Duplicates of whole blood corresponding to 10 mg of Fe were pre-treated and finally analysed in a liquid scintillator (Tri-Carb; Packard Instruments) to determine the radiation from ^55^Fe and ^59^Fe.

Iron absorption is affected not only by the composition of the meal but also by intra-individual differences in iron absorption capacity, in which iron status is the predominant affecting factor^(^ ^25^ ^–^ ^27^ ^)^. This entails a problem when comparing different meals administered to subjects with different iron status and different iron absorption capacity, and thus there was an informal agreement on normalising iron absorption results to the 40 % absorption from a reference dose of iron. The normalised meal absorption is the iron absorption for an individual having a reference dose absorption of 40 %^(^ ^27^ ^)^, corresponding to borderline iron-deficit individuals who have not developed anaemia. The absorbed amount of iron at this standardised iron status is obtained by multiplying the drink and reference dose ratio by 40.

### Composition of the fruit drink

The fruit drink with an oat base included grape, mango, passion fruit, banana and added sugar. Mango provided 6·5 %, the other fruits provided 28·5 %, oat base provided 5 % and added sugar provided 4 % of the content. The fruit drinks were supplemented with iron (2·1 mg/100 ml, in the form of ferrous lactate dehydrate; Vitablend) and ascorbic acid (50 mg/100 ml; Vitablend) and were produced in connection with Trial 1 and Trial 2, respectively. Lp299v was added as a fermented oat base, and the products were balanced so that they all contained the same amount of oat base (5/100 ml) by adding heat-treated fermented oat base (5 ml in control product, 4·5 ml in low-dose 299v product). The composition of the study drinks is presented in Table 1. Lp299v was analysed according to Johansson *et al*.^(^ ^28^ ^)^. Total iron was determined by the method of Björn-Rasmussen *et al*.^(^ ^29^ ^)^ and PA by the method described by Harland & Oberleas^(^ ^30^ ^)^. Lactate and acetate were analysed with an enzymatic and spectrophotometric method (Boehringer Mannheim/R-Biopharm AG). The drink with the lower dose of Lp299v (1·3×10^9^ CFU/200 ml) contained similar amounts of all nutrients compared with the control drink, whereas the drink with the higher dose of Lp229v (1·7×10^10^ CFU/200 ml) had a somewhat higher lactate and acetate content. The appearance, taste and texture of all study products were identical.

**Table 1:** Composition of the study products (per 200-ml drink)

	Trial 1		Trial 2	
	Fruit drink with Lp299v	Control fruit drink	Fruit drink with Lp299v	Control fruit drink
Lp299v (CFU)	1·3×10^9^	ND*	1·7×10^10^	ND*
Iron (mg)†	4·6	5·2	5·2	5·4
PA (mg)	3·4	3·4	3·2	3·8
PA:Fe molar ratio	0·06	0·05	0·05	0·06
d-Lactate (mmol)	0·34	0·36	1·64	0·42
l-Lactate (mmol)	0·44	0·44	1·40	0·66
Acetate (mmol)	0·06	0·06	0·50	0·16
AA (mg)	100	100	100	100
AA:Fe molar ratio	6·9	6·1	6·1	5·9
Energy (kJ)	376·5	376·5	376·5	376·5
Energy (kcal)	90	90	90	90

Lp299v, *Lactobacillus plantarum* 299v; CFU, colony-forming units; ND, not detected; PA, phytic acid; AA, ascorbic acid.

* <10 CFU/ml.

† Of which 4·2 mg as ferrous lactate dehydrate.

### Ethics

The study was conducted according to the Ethical Principles for Medical Research Involving Human Subjects, adopted by the 18th World Medical Association General Assembly, in Helsinki, Finland, in June 1964. The study protocol was approved by the Ethics Review Board in Gothenburg (Registration Diary number: 181-07), as well as by the Radiation Protection Committee and Radiation Ethics at the Sahlgrenska University Hospital (Diary number: 07-13). All subjects were given detailed information, both in written form and orally, about the purpose and procedures of the study. Consent to participate was signed by the subject before the study started, and the subject was free to withdraw from the study at any time without giving any explanation.

### Subjects

The study population consisted of twenty-two healthy Swedish women of reproductive age recruited from students at the University of Gothenburg.

### Inclusion/exclusion criteria

The subjects had to be healthy women of reproductive age not on medication (with the exception of oral contraceptives) or with any gastrointestinal, malabsorptive or metabolic diseases. They should not be pregnant or lactating, and they should not have donated blood within 2 months before the study. They should not have taken any dietary supplements (including iron) during the study, nor within the 2 weeks before the study. Exclusion criteria also included infection/inflammation, as an activated acute-phase reaction has a marked effect on iron homoeostasis and iron absorption

### Laboratory analysis

Blood samples were collected by venepuncture directly after the whole-body counting, which took place about 14 d after the intake of the radio-iron-labelled fruit drinks. Serum iron concentration, total iron-binding capacity, transferrin saturation, S-ferritin, soluble transferrin receptor and Hb and C-reactive protein (CRP) were analysed at an accredited reference laboratory (Clinical Chemistry Laboratory, Sahlgrenska University Hospital), according to ISO/IEC 15189 Standard for Medical Laboratories.

### Infection assessment

CRP and erythrocyte sedimentation rate were analysed in order to avoid systematic error introduced by infections. Before being served the drinks, each subject was asked about the present health status and any indications of infection during the weeks before drinking the radio-iron-labelled fruit drinks. The same question was also asked in connection with the blood sampling.

### Statistics

The primary hypothesis was that there would be a significant increase in iron absorption after drinking a fruit drink containing Lp299v. The sample size and power calculation was based on the fact that two different iron isotopes were used in a cross-over design, making each subject their own control, thereby allowing the use of the paired Student’s *t* test. On the basis of the content, the iron absorption from the control fruit drink was expected to be approximately 20 %^(^ ^5^ ^)^. Using a two-sided paired *t* test and with a significance level of 0·05, ten subjects would be needed in order to obtain 90 % probability (i.e. a power of 90 %) of observing a 7·5(sd 5·0) % difference in iron absorption. Descriptive data are presented as mean values and standard deviations. Data were checked for normality of distribution using the Shapiro–Wilk test. The paired sample Student’s *t* test, at a 95 % CI, was used to analyse iron absorption differences between Lp299v-containing fruit drink and control in the same trial. The unpaired two-sample Student’s *t* test was used to analyse differences between Lp299v-containing fruit drinks in Trials 1 and 2, as well as between control drinks in Trials 1 and 2. As a sensitivity analysis, the non-parametric counterparts (Wilcoxon signed rank sum test and Mann–Whitney test) were also conducted, but it did not change the outcome. All *P*-values are two-tailed and considered to be statistically significant if *P*<0·05. Statistical analyses were performed using IBM^®^ SPSS^®^ statistics for Windows 22.0.0.0 (IBM Corp.).

## Results

**Fig. 2.**
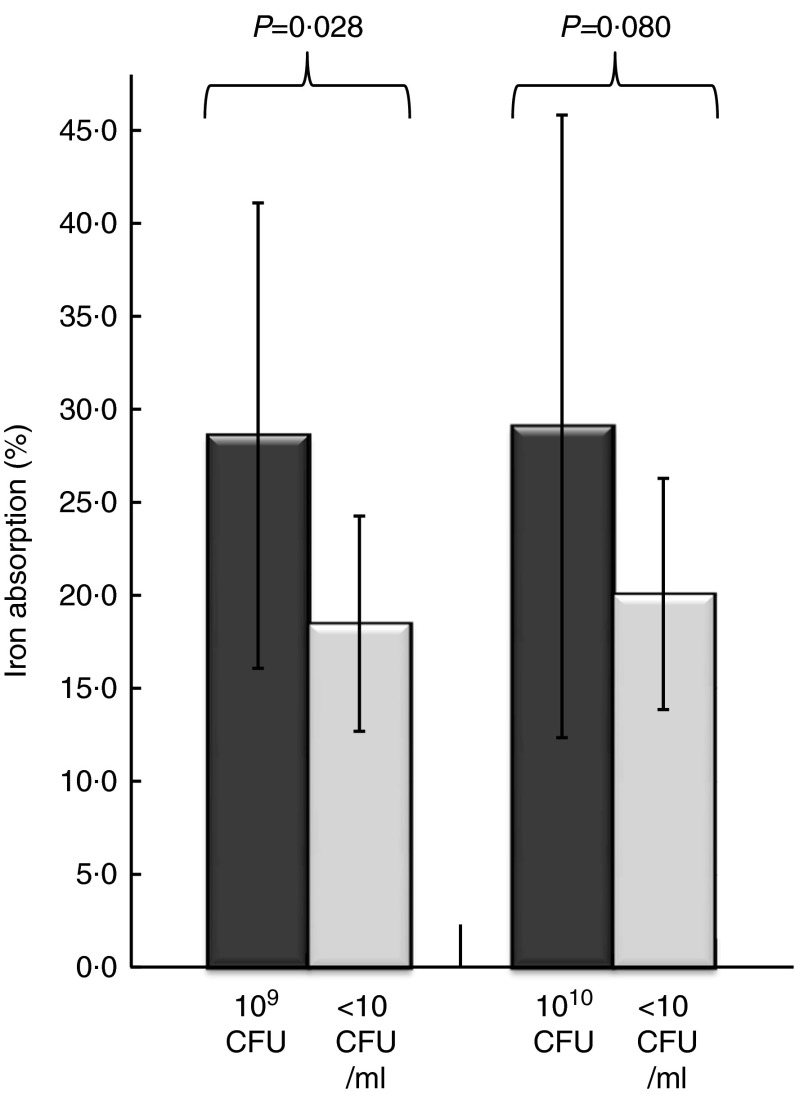
Iron absorption from a fruit drink with and without *Lactobacillus plantarum* 299v (Lp299v). The bars and the whiskers represent means and standard deviations for non-haem iron absorption. The iron absorption values are normalised to 40 % absorption of the reference dose. The lactobacilli fruit drink in Trial 1 contained 10^9^ colony-forming units (CFU) Lp299v (*n* 10) and the lactobacilli fruit drink in Trial 2 contained 10^10^ CFU Lp299v (*n* 11). A paired sample Student’s *t* test was used to analyse iron absorption differences between Lp299v-containing fruit drink and control in the same trial.

The presented iron absorption values were normalised to a 40 % reference dose absorption, and thereby corrected for variation in iron absorption capacity that mainly results from differences in iron status. Of the twenty-two subjects, one subject did not complete the study so that the data analysis was based on a total of twenty-one women with an age range of 20–40 years (mean age=24·3 years) and a mean BMI of 21·2 (sd 2·1) kg/m^2^ (Table 2). The iron absorption values (Fig. 2) in both trials were distributed according to the Gaussian curve.

**Table 2:** Study subject data at baseline (Mean values and standard deviations)

	Trial 1 (10^9^ CFU Lp299v, *n* 10)		Trial 2 (10^10^ CFU Lp299v, *n* 11)		Total (*n* 21)		
	Mean	sd	Mean	sd	Mean	sd	*P* *
BMI (kg/m^2^)	21·3	2·0	21·2	2·3	21·2	2·1	NS
S-ferritin (μg/l)	33	13	33	14	33	13	NS
Hb (g/l)	138	8	135	9	136	9	NS
S-Fe (μmol/l)	15	6	18	7	17	7	NS
TSAT (%)	23	10	2	9	23	9	NS
TIBC (μmol/l)	69	7	76	10	73	9	NS
CRP (mg/l)	<5		<5		<5		NS
ESR (mm)	9	5	7	3	8	4	NS

CFU, colony-forming units; Lp299v, *Lactobacillus plantarum* 299v; S-Fe, serum iron concentration; TSAT, transferrin saturation; TIBC, total iron-binding capacity; CRP, C-reactive protein; ESR, erythrocyte sedimentation rate.

* *P* values represent differences between Trials 1 and 2. Values are considered to be statistically significant if *P*<0·05.

### Trial 1

The results of Trial 1 involved ten subjects. Mean iron absorption from the drink containing 10^9^ CFU Lp299v was 28·6(sd 12·5) %, which was significantly higher than the iron absorption from the control drink, 18·5(sd 5·8) % (*P*<0·028).

### Trial 2

The results of Trial 2 involved eleven subjects. Mean iron absorption from the drink containing 10^10^ CFU Lp299v was 29·1(sd 17·0) % compared with 20·1(sd 6·4) % from the control drink. However, this difference was not statistically significant (*P*<0·080).

Analysis of the iron absorption from the two drinks containing Lp299v in Trials 1 and 2 showed that they did not differ significantly (*P*<0·941), nor did the iron absorption from the two control drinks in Trials 1 and 2 differ (*P*<0·557). Combining the two studies by pooling the absorption values for all twenty-one subjects gave a mean iron absorption of 28·8(sd 14·7) % with added Lp299v, which was significantly higher than the control drink iron absorption (19·3(sd 6·0) %, *n* 21, *P*<0·004).

## Discussion

In this study, the absorption of non-haem iron from a fruit drink containing Lp299v was almost 50 % higher compared with a similar fruit drink without Lp299v. The iron absorption from the control drink was 19 % (pooled value for Trials 1 and 2, *n* 21), and thus the drink can be considered to be a high iron bioavailability meal^(^ ^4^ ^)^. The high iron bioavailability was probably because the drinks contained 100 mg of ascorbic acid (AA). The molar ratio of AA to iron in the control drink was about 6:1, and an enhancing effect on iron absorption has been proposed at molar ratios of at least 2:1 to 5:1 (depending on the amount inhibiting factors)^(^ ^31^ ^,^ ^32^ ^)^. Nevertheless, when Lp299v was added to this drink with an already relatively high absorption level, there was a significant increase in iron absorption. In a study by Bering *et al*.^(^ ^17^ ^)^, where the study product (an oat gruel) had a low iron bioavailability, adding Lp299v resulted in an approximately 100 % increase in iron absorption in comparison with the present study with a high bioavailability meal in which the relative increase was 50 %. Hallberg *et al*.^(^ ^33^ ^)^ similarly observed a proportionally greater influence of AA on low iron bioavailability meals.

In the present study, iron absorption from the fruit drink containing 10^9^ CFU Lp299v did not differ from the fruit drink with 10^10^ CFU Lp299v. Consequently, the iron-enhancing effect did not seem to be augmented when the Lp299v concentration was increased from 10^9^ to 10^10^ CFU. The drink with a higher content of Lp299v contained somewhat more organic acids, and this could theoretically have a positive effect on the iron absorption^(^ ^17^ ^,^ ^34^ ^,^ ^35^ ^)^. However, it is unclear to what extent organic acids such as lactate and acetate promote iron absorption (*Nordic Nutrition Recommendations 2012*), and the differences between the Lp299v drinks were small (2 mmol and approximately 0·4 mmol, respectively).

Even if the fruit drink with the highest dose of Lp299v contained more organic acids, no statistical significant difference in iron absorption was detected between the control drink and the Lp299v drink (*P*<0·080). However, it cannot be ruled out that the lack of significant difference could be because of the sample size being too small.

Previous studies on iron absorption and iron status have also suggested positive effects from lactobacilli. In an Indian population, the consumption of *Bifidobacterium lactis* HN019 and prebiotic fortified milk for 1 year resulted in a smaller number of iron-deficient preschoolers and increased weight gain^(^ ^36^ ^)^.

It is well known that probiotics can contribute to, and modulate, health and disease^(^ ^37^ ^)^, but little is known about a possible iron absorption-enhancing mechanism(s). Some *Lactobacillus* strains have inherent phytases and can degrade PA during fermentation, thereby making the bound iron available for absorption^(^ ^38^ ^)^. However, Lp299v has low phytase activity, and the amount of PA is not lowered during fermentation of PA-rich oat gruel in comparison with non-fermented oat gruel^(^ ^17^ ^)^. The content of PA was also rather low in the fruit drinks used in this study; all drinks contained the same amount of PA and the PA:Fe molar ratio was very low (0·05–0·06). Furthermore, even if all of the PA was degraded in the intestine, only a small amount of iron should be released (0·3 mg). This is calculated from Bering *et al*.^(^ ^17^ ^)^ assuming that all of the iron in the oat gruel meal was bound to PA.

Bering *et al*.^(^ ^17^ ^)^ studied the effect of pH and organic acids on the absorption of iron in the presence of Lp299v in human trials involving women of reproductive age. In this study, Lp299v-fermented oat gruel was compared with three control products: heat-treated Lp299v-fermented oat gruel, non-fermented oat gruel with the same pH as the fermented oat gruels and non-fermented gruel with organic acids (similar amount as in the fermented gruels). The iron absorption from the Lp299v-fermented oat gruel was significantly higher compared with all the other control products. This led the authors to conclude that the positive effect on iron absorption was a result of the live Lp299v, and not the fermentation *per se*, pH or by adding organic acids.

The improvement in iron absorption may be related to the colonisation of Lp299v in the intestine. A mannose adhesion-encoding gene in *L. plantarum* has been identified^(^ ^39^ ^)^, and it has been shown that Lp299v can adhere to the intestinal epithelium via a mannose-binding mechanism^(^ ^40^ ^)^. Tallon *et al*.^(^ ^41^ ^)^ showed that Lp299v can also adhere to mucin that covers the epithelium in the intestine, and *in vitro* trials indicate that Lp299v increases the mucin excretion^(^ ^42^ ^)^. Mucin may be involved in iron absorption. Conrad *et al*.^(^ ^43^ ^)^ showed that mucins can bind iron and that these mucin–iron complexes prevent precipitation of the iron. Divalent metal transporter 1, which can transport Fe2+ into the cells, is concentrated in mucin vesicles near the luminal surface, strengthening the role of mucin in iron uptake^(^ ^44^ ^)^.

Another possible mechanism underlying the observed results could be an increase in colonic iron absorption due to a decrease in colonic pH, thereby reducing ferric iron into highly absorbable ferrous iron as a result of lactobacilli growth. In a cell-line study by Bergqvist *et al*., lactic acid fermentation by *Lactobacillus* enhanced Caco-2 iron uptake from carrot juice. As soluble ferrous iron was increased about 16-fold by lactic acid fermentation, and about one-third of the ferrous iron remained soluble after *in vitro* digestion (about 4- to 5-fold higher than in fresh juice), the authors concluded that enhanced iron uptake was a result of the increased level of soluble ferrous iron^(^ ^45^ ^)^. However, a study by Petry *et al*.^(^ ^46^ ^)^ showed that although inulin administration decreased faecal pH and increased faecal *Bifidobacterium* it did not influence iron absorption. Furthermore, in the study by Bering *et al*.^(^ ^47^ ^)^, no absorption of non-haem iron in the distal part of the intestine (ileum, colon) was observed in healthy young women.

In the present study, as in every study, there are several potential sources of error, both random and systematic, which ought to be minimised. In iron absorption studies, random sources of error are present in everything from preparation of isotopic solutions and meals to the very handling of the blood samples. This has previously been discussed thoroughly^(^ ^48^ ^)^. The main limitations of the present study could, as discussed earlier regarding the trial with the high dose of Lp299v, include the small sample size. In addition, iron absorption capacity for women can differ according to the menstrual cycle phase. As all subjects in the present study were women, the optimal approach would therefore be to administer the reference dose and the fruit drinks at the same point in the menstrual cycle – that is, 4 weeks apart. This would ensure that subjects had a similar absorption capacity when administering both the fruit drinks and the reference dose. However, even if there had been a large difference in iron absorption capacity at the two points in time, it would have had no impact on the present results. The only consequence would be difficulties when comparing present results with results in other studies. The ratio (i.e. difference) between the Lp299v drink and the control drink remains regardless.

Another limitation was that blood for analysis of the iron and inflammatory status was only drawn 2 weeks following the fruit drink administration. To gain a better overall insight into iron and inflammatory status, blood samples should also have been drawn when the fruit drinks were administered.

The source of error with the largest impact is the compliance of the subjects, which includes heeding the instructions regarding fasting when served the study meals and the reference dose.

As a consequence of the observed significant iron absorption-enhancing effect of Lp299v, there might be practical applications of such lactobacilli products in populations with high prevalence of iron deficiency (e.g. developing countries). However, supplementing food stuffs with live probiotics results in difficulties with regard to storage and shelf life. This could, in theory, be overcome by using lyophilised probiotics, which could either be administered in capsules or as sprinkles in fortification programmes. However, it is still unknown whether the iron absorption-enhancing properties of Lp299v remain following direct administration of lyophilised probiotics.

As can be seen from Fig. 2, the iron absorption from the fruit drinks with Lp299v had a much wider distribution than the control drinks. The interquartile range for iron absorption in Trial 1 from the Lp299v drink was 18·7 % and from the control drinks it was 8·2 %, respectively. The corresponding interquartile ranges in Trial 2 were 21·7 and 8·9 %, respectively. An interpretation is that the subjects when consuming an Lp299v drink had a much more diverse response compared with when they merely drank a control drink without Lp299v. In fact, six out of the twenty-one subjects did not respond positively to the added Lp299v.

It has been estimated that approximately 1200 different bacterial species inhabit the human gastrointestinal tract, and that each individual harbours at least 160 of these species^(^ ^49^ ^)^. Interestingly, in iron-deficient and anaemic women in southern India, it was observed that the amount of faecal lactobacilli were significantly lower than in a control group. However, there was no difference between the two groups with respect to the other investigated bacteria strains^(^ ^50^ ^)^. With this in mind, the diverse response to the addition of Lp299v in the present study could perhaps be found in an intra-individual difference in the already existing microbiota. If this were the case, adding Lp299v to a microflora already rich in Lp299v-related lactobacilli should not give any further effect compared with if it were added to a gastrointestinal flora with little lactobacilli.

Interestingly, the reference dose absorption (which is used as a comparable measure of iron absorption capacity, and thereby facilitates comparisons between different studies) in the six individuals not responding positively to the added Lp299v in this present study was higher (mean Fe abs=35·4 %) compared with the fifteen individuals who did actually respond (mean Fe abs=29·7 %). However, the small sample size (*n* 6) probably prevented the difference in reference dose absorption being statistically significant. Nevertheless, the question of whether this was because of a gastrointestinal environment already rich in Lp299v could not be answered in this present study. Future studies involving faecal sampling could cast more light on these questions.

In conclusion, intake of Lp299v can increase iron absorption by an additional 50 % from a fruit drink that already has a relatively high iron bioavailability. This effect may be related to the colonisation of the bacteria in the intestine.
